# Coffee consumption and cancer risk: a Mendelian randomisation study

**DOI:** 10.1016/j.clnu.2022.08.019

**Published:** 2022-08-25

**Authors:** Paul Carter, Shuai Yuan, Siddhartha Kar, Mathew Vithayathil, Amy M Mason, Stephen Burgess, Susanna C Larsson

**Affiliations:** 1 Department of Medicine, University of Cambridge, Cambridge, United Kingdom; 2 Unit of Cardiovascular and Nutritional Epidemiology, Institute of Environmental Medicine, Karolinska Institutet, Stockholm, Sweden; 3 MRC Integrative Epidemiology Unit, Bristol Medical School, University of Bristol, Bristol, United Kingdom; 4 MRC Cancer Unit, University of Cambridge, Cambridge, United Kingdom; 5 British Heart Foundation Cardiovascular Epidemiology Unit, Department of Public Health and Primary Care, University of Cambridge, Cambridge, UK; 6 National Institute for Health Research Cambridge Biomedical Research Centre, University of Cambridge and Cambridge University Hospitals, Cambridge, UK; 7 MRC Biostatistics Unit, University of Cambridge, Cambridge, United Kingdom; 8 Department of Public Health and Primary Care, University of Cambridge, Cambridge, United Kingdom; 9 Department of Surgical Sciences, Uppsala University, Uppsala, Sweden

**Keywords:** Coffee, Cancer, Mendelian randomization

## Abstract

**Background:**

Coffee contains many bioactive chemicals and associations with cancer have been reported in observational studies. In this Mendelian randomisation (MR) study we investigated the causal associations of coffee consumption with a broad range of cancers.

**Materials and Methods:**

Twelve independent genetic variants proxied coffee consumption. Genetically-predicted risk of any cancer (59,647 cases) and 22 site-specific cancers was estimated in European-descent individuals in UK Biobank. Univariable and multivariable MR analyses were conducted.

**Results:**

Genetically-predicted coffee consumption was not associated with risk of any cancer in the main analysis (OR 1.05, 95% CI 0.98-1.14, p=0.183) but was associated with an increased risk of digestive system cancer (OR 1.28, 95% CI 1.09-1.51, p=0.003), driven by a strong association with oesophageal cancer (OR 2.79, 95% CI 1.73-4.50, p=2.5×10^-5^). This association was consistent after adjustment for genetically-predicted body mass index, smoking and alcohol consumption. There was no strong evidence supporting a causal relationship between genetically-predicted coffee consumption and the majority of cancers studied. However, genetically-predicted coffee consumption was associated with increased risk of multiple myeloma (OR 2.25, 95% CI 1.30-3.89, p=0.004) and reduced ovarian cancer risk (OR 0.63, 95% CI 0.43-0.93, p=0.020).

**Conclusions:**

This MR study provides strong support for a causal association of coffee consumption with oesophageal cancer, but not for the majority of cancer types, and the underlying mechanisms require investigation.

## Introduction

Coffee is one of the most widely consumed beverages worldwide, with an estimated 165 million 60 kg bags consumed per year (1). The potential public health impact of coffee drinking is therefore substantial, and effects on health have been suggested including on cardiovascular disease, type 2 diabetes, liver conditions, and all-cause mortality (2). Cancer risk is increasingly being found to be influenced by dietary factors and coffee contains over 1000 bioactive compounds with antioxidant, anti-inflammatory and antifibrotic properties, with the potential to influence carcinogenesis. Many of such compounds have been reported to promote anticarcinogenic effects including kahweol (3), polyphenols (4) and caffeine (5).

Coffee consumption and cancer risk has been investigated in a number of epidemiologic studies. Although protective associations have been reported with multiple cancer types, including colorectal (6), prostate (7), liver (8) and endometrial (9) cancers, overall findings have been discordant in terms of all cancer risk and site-specific cancers (2,10). The oncological effects of coffee drinking therefore remain equivocal, as reflected in the latest report of the International Agency for Research on Cancer (IARC) in 2016 which evaluated coffee as unclassifiable as to its carcinogenicity in humans, although they concluded that drinking very hot beverages likely promotes oesophageal carcinogenesis through a temperature effect (11). The current evidence base is lacking due to difficulties with observational epidemiological studies which are influenced by reverse causality and confounding, which is particularly relevant to coffee drinking which may be associated with other lifestyle behaviours.

Mendelian randomisation (MR) is an epidemiological approach with the potential to avoid such biases. The technique assesses whether the genetically-predicted levels of a risk factor (such as coffee drinking) and a disease outcome (such as cancer) are associated. As Mendel’s law of independent assortment states that characteristics are inherited independently of each other, these associations are less susceptible to confounding. Furthermore, as genetic variants are established from birth, the potential for reverse causality is diminished. Therefore, associations in the MR study are more likely to have a causal interpretation than those from conventional epidemiological analyses. Thus far, only a limited number of MR studies have assessed the association between coffee drinking and cancer with no association found with overall cancer (12), colorectal (13), breast (14), prostate (15) and ovarian (16) cancers. Such studies are informative, but, the majority have not adjusted for important factors associated genetically with coffee consumption, such as body mass index (BMI) and smoking habits. Furthermore, most studies only focused on individual cancers, and the causal association with many highly prevalent cancer types remains unstudied.

With this in mind, the primary aim of this study was to use an MR approach to investigate the associations of genetically-predicted coffee consumption and the risk of cancer overall, and of 22 site-specific cancers. We used data from UK Biobank and four large-scale genome-wide association studies consortia. In complementary analyses we adjusted the results for BMI, smoking initiation, and alcohol consumption, which are genetically associated with coffee consumption.

## Materials and Methods

### Study design

The present study was based on published genome-wide association studies (GWASs), and the UK Biobank study. We first investigated the associations of genetically proxied coffee consumption with any cancer and site-specific cancers in UK Biobank. Given genetic associations of coffee consumption with BMI and smoking behaviours, we further used multivariable MR design to systematically minimize the pleiotropy from these two traits. All included GWASs were approved by corresponding ethics committees and participants provided written informed consent. The present analyses were approved by the Swedish Ethical Review Authority.

### SNP selection

Fifteen single nucleotide polymorphisms (SNPs) were identified to be associated with coffee consumption at the genome-wide significance level from a meta-analysis of GWASs on habitual coffee consumption (17). The meta-analysis included a discovery stage based on UK Biobank (n=85 852) and a replication stage including Nurses’ Health Study (n = 10 675), Health Professionals Follow-up Study (n = 6618) and Women’s Genome Health Study (n = 22 691) (17). We estimated genetic association across 15 SNPs using European samples of 1000 genomes data as the reference panel (18). Twelve independent SNPs (*r*^2^ <0.01 and clumping distance >10,000kb) were employed as genetic instruments for coffee consumption after removal of rs4719497, rs12699844 and rs117692895 due to correlation with other selected variants. Information on the SNPs used to proxy coffee consumption is provided in Table 1. Most SNPs were in gene regions that likely act directly on coffee drinking behaviour or affect drinking behaviour indirectly by altering the metabolism of caffeine and thus caffeine levels (19). One SNP (rs597045) is located near a locus linked to smell/taste perception of coffee (19). A previous investigation has shown that several of these genetic variants are also associated with consumption of hot drinks other than coffee (20), meaning that the results of this investigation relate to hot drink consumption more generally and not coffee specifically. Table 1 also shows details for two SNPs used in analyses of genetically-predicted caffeine consumption.

### Data source of cancer

Genetically-predicted risk of cancer at 22 sites, any cancer (one of the 22 sites combined), any digestive system cancer, and any non-digestive system cancer were estimated in the UK Biobank (21). This cohort study recruited around 500 000 adults, aged 37 to 73 years, across the UK during 2006 to 2010. To reduce population stratification bias, we confined the study population to European-descent individuals. After exclusion of related individuals (third-degree relatives or closer), low call rate, and excess heterozygosity (3 or more standard deviations from the mean), 367,643 participants remained in the analyses and were followed up until March 31, 2017 or death. Findings for cancers with evidence of association with coffee consumption in UK Biobank (p<0.05) were tested for replication in the FinnGen consortium using the R5 release (n=218,792) (22).

### Data sources of BMI, smoking initiation, and alcohol consumption

Analyses were adjusted for differences in genetically-predicted BMI, smoking initiation and alcohol consumption using multivariable MR. Summary-level data for BMI was available from a meta-analysis of GWASs for body fat distribution in 694,949 patients of European Ancestry, as described previously (23). Summary-level data for smoking initiation was available from GWAS of 1,232,091 individuals, as described previously (24). Summary-level data for alcohol consumption was available from GWAS of 941,280 individuals, as described previously (24).

### Statistical analysis

The inverse-variance weighted median method with random-effects was used in the main analyses. Two sensitivity analysis, including the weighted median and MR-Egger, were utilized to examine the consistency of results and detect and correct for directional pleiotropy. The effect sizes of the associations between genetically predicted coffee consumption and cancer risk were scaled to a 50% increase in coffee consumption. All analyses were two-sided and performed using the mrrobust package (25)in Stata/SE 15.0 and MendelianRandomization (26)and TwoSampleMR (27) packages in R Software 3.6.0.

## Results

### Any Cancer

Of 367,561 European-descent participants, 59,647 had one of the 22 site-specific cancers included in the study, and formed the any cancer group. Genetically predicted coffee consumption was not associated with risk of any cancer in the main analysis (odds ratio [OR] 1.05, 95% confidence interval [CI] 0.98-1.14, p=0.183) or after adjustment for genetically predicted BMI (OR 1.06, 95% CI 0.97-1.16, p=0.193), smoking (OR 1.06, 95% CI 0.98-1.14, p=0.143), or alcohol consumption (OR 1.08, 95% CI 0.97-1.19, p=0.156).

### Digestive System Cancer

**Fig 7:**
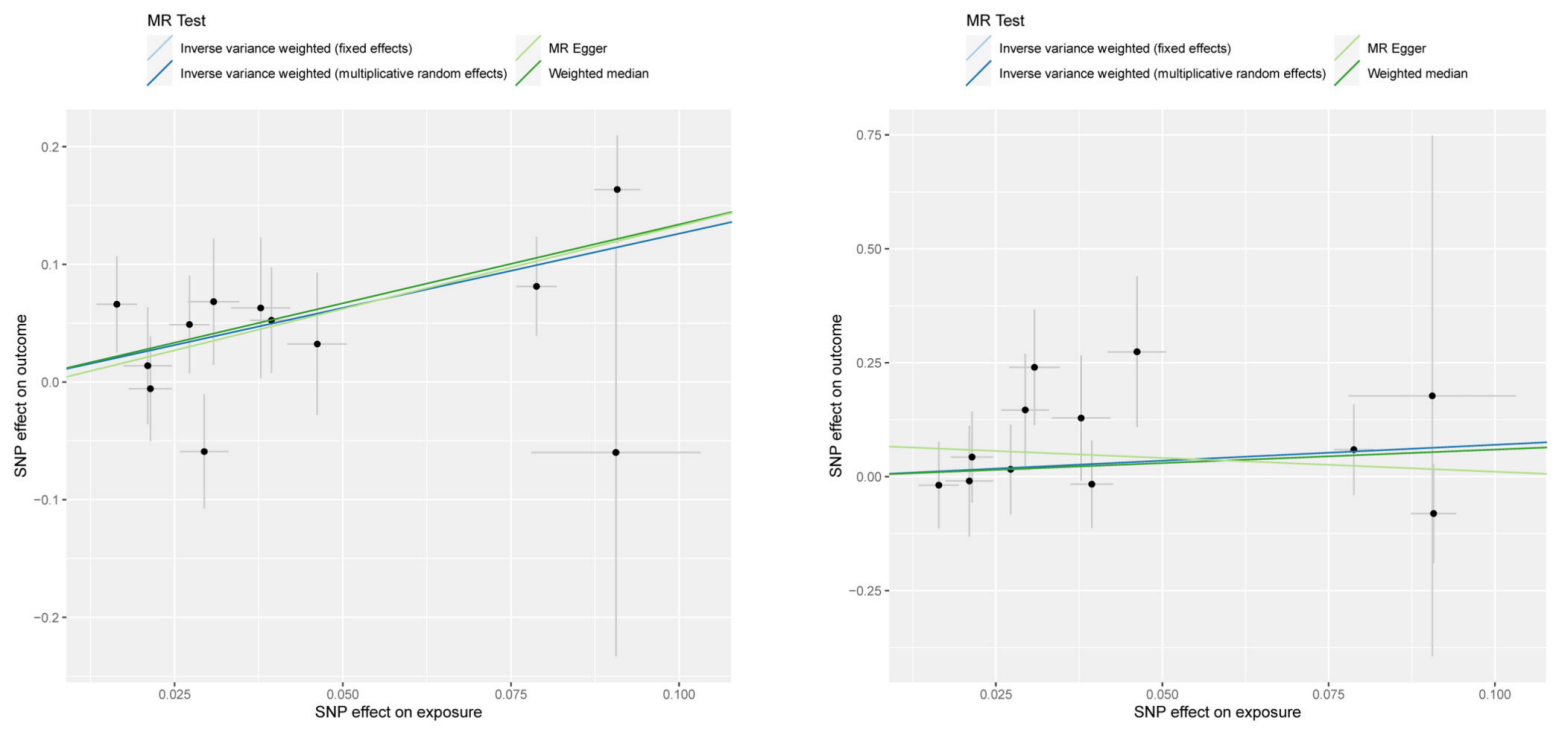
Scatter plots for Mendelian randomization analysis of coffee consumption and risk of oesophageal cancer (A) associations in UK BioBank (B) associations in FinnGen. Horizontal axis: SNPs’ association with coffee consumption. Vertical axis: SNPs’ association with oeseophageal cancer.

Genetically predicted coffee consumption was associated with an increased risk of digestive system cancer (OR 1.28, 95% CI 1.09-1.51, p=0.003). This finding remained similar in multivariable MR analysis with adjustment for genetically predicted BMI (OR 1.23, 95% CI 1.02-1.49, p=0.031), smoking initiation (OR 1.27, 95% CI 1.08-1.50, p=0.004), and alcohol consumption (OR 1.34, 95% CI 1.07-1.68, p=0.010). Risk of digestive cancer was driven by a strong association with oesophageal cancer in UK Biobank (OR 2.79, 95% CI 1.73-4.50, p=2.5×10^-5^) which was consistent after adjustment for genetically predicted BMI (OR 3.22, 95% CI 1.84-5.63, p=4.1×10^-5^), smoking (OR 2.77, 95% CI 1.71-4.88, p=3.3×10^-5^), and alcohol consumption (OR 2.98, 95% CI 1.56-5.70, p=0.001), and across sensitivity analyses (Supplemental table 2). In the FinnGen consortium, genetically predicted coffee consumption was also associated with a large magnitude estimate of increased oesophageal cancer risk (OR 2.01, 95% CI 0.57-7.05, p=0.278). This association did not reach statistical significance, likely due to the limited number of oesophageal cancers (n=232) and although it was similar after adjustment for smoking initiation, it was attenuated after adjustment for BMI. Scatter plots for mendelian randomization analysis of coffee consumption and risk of oesophageal cancer are shown in figure 7. Otherwise, there was suggestive evidence of an association between genetically predicted coffee consumption and risk of pancreatic cancer in the main analysis (OR 1.40, 95% CI 0.94-2.10, p=0.097) and after adjustment for genetically predicted BMI (OR 1.29, 95% CI 0.81-2.06, p=0.291), smoking (OR 1.37, 95% CI 0.92-2.05, p=0.126), and alcohol consumption (OR 1.52, 95% CI 0.88-2.62, p=0.133).

### Non-Digestive System Cancer

There was no evidence for an association between genetically predicted coffee consumption and risk of non-digestive system cancer overall (OR 1.01, 95% CI 0.93-1.10, p=0.820). However, coffee consumption was associated with an increased risk of multiple myeloma in UK Biobank in the main analysis (OR 2.25, 95% CI 1.30-3.89, p=0.004), and the association remained after adjustment for genetically predicted BMI (OR 2.61, 95% CI 1.37-4.96, p=0.003),smoking (OR 2.25, 95% CI 1.29-3.90, p=0.004) and alcohol consumption (OR 2.81, 95% CI 1.34-5.93, p=0.006). The positive association with multiple myeloma was not replicated in the FinnGen consortium. Genetically predicted coffee consumption was associated with a consistent reduction in risk of ovarian cancer in UK Biobank in the main analysis (OR 0.63, 95% CI 0.43-0.93, p=0.020) and after adjustment for genetically predicted BMI (OR 0.59, 95% CI 0.38-0.94, p=0.026), smoking (OR 0.62, 95% CI 0.42-0.92, p=0.017) or alcohol consumption (OR 0.54, 95% CI 0.32-0.92, p=0.024). The inverse association with ovarian cancer was not replicated in the FinnGen consortium. There was suggestive evidence of an inverse association between genetically predicted coffee consumption and risk of leukaemia (OR 0.70, 95% CI 0.47-1.03, p=0.069) and prostate cancer (OR 0.85, 95% CI 0.72-1.01, p=0.070).

### Stratified analyses of coffee type and temperature preference and risk of oesophageal cancer

Due to robust findings of increased oesophageal cancer risk with genetically predicted coffee consumption we conducted post-hoc stratified analyses to investigate possible heterogeneity in associations depending on coffee drinking preferences (Supplemental table 3). Increased risk of oesophageal cancer was consistently associated with genetically predicted coffee consumption across individuals with preference for warm (OR 2.74, 95% CI 1.34-5.60, p=0.06), hot (OR 5.45, 95% CI 1.37-21.7, p=0.016) and very hot drinks (OR 4.09, 95% CI 0.68-24.7, p=0.125) although the latter did not reach statistical significance. Analyses stratified by self-reported coffee consumption demonstrated that increased oesophageal cancer risk was similar in individuals reporting 1-3 cups per day (OR 4.24, 95% CI 1.9-9.45, p<0.001) and in those reporting no coffee consumption (OR 4.23, 95% CI 1.21-14.9, p=0.024). Given the high prevalence of tea consumption in the UK, it is likely that many of those reporting no coffee consumption were tea drinkers. Analyses stratified by caffeine preference demonstrated enhanced risk of oesophageal cancer with genetically predicted coffee consumption amongst those with a preference for caffeinated drinks (OR 4.75, 95% CI 2.28-9.86, p<0.001) but not amongst drinkers of decaffeinated coffee (OR 1.01, 95% CI 0.22-4.6, p=0.989).

### Caffeine consumption

As caffeine is a major bioactive compound in coffee we performed MR analyses of genetically-predicted caffeine consumption using a genetic instrument consisting of 2 SNPs associated with caffeine consumption (Table 1). Similarly, to coffee, genetically-predicted caffeine consumption was positively associated with an increased risk of GI cancer (OR 1.17, 95% CI 1.01-1.35, p=0.039), oesophageal cancer (OR 2.20, 95% CI 1.43-3.4, p=3.4×10^-4^) and multiple myeloma (OR 1.77, 95% CI 1.08-2.91, p=0.025 but inversely associated with risk of leukaemia (OR 0.67, 95% CI 0.47-0.96, p=0.030).

## Discussion

Our main finding of a causal association of coffee drinking with risk of digestive system cancer has been previously suggested, although this has not been supported by previous observational studies (28). Furthermore, protective associations of coffee with multiple digestive system cancer types including colorectal, oesophageal, pancreatic and hepatocellular carcinomas have previously been reported observationally (29,30). As lifestyle behaviours are prone to reverse causality and confounding, the MR approach we used provides stronger evidence regarding causality. In line with this, Ong et al found a positive association between coffee intake and colorectal cancer risk in UK Biobank using an observational approach which was abolished when they used a stringent genetic instrument for coffee consumption (12). This is one of only two previous MR analyses of coffee consumption and digestive system cancer risk, with both focused on colorectal cancer specifically. Consistent with our findings, Cornish et al also found no genetic association of only 4 SNPs related to coffee consumption and risk of colorectal cancer in UK Biobank(13). Taken together, the present MR study and prior literature do not support a role of coffee in influencing carcinogenesis of colorectal cancer and a broader range of digestive system cancers.

Augmented risk of oesophageal cancer was the main determinant of the increased digestive system cancers with coffee drinking reported in the present study. We provide strong evidence for a causal relationship which is large in magnitude (3-fold) and consistent across sensitivity analyses and in a replication study. Previous observational studies do not support this but were generally based on small sample sizes with only few cases. In a systematic review and meta-analysis of 11 studies assessing the association between coffee consumption and oesophageal cancer, five studies reported an effect size estimate (odds ratio or relative risk) above 1 and six studies an effect size estimate below 1, with an overall inverse association in East Asians but not in Euro-American populations (31). These divergent findings may relate to imprecise estimates, varying proportions of different histological subtypes of oesophageal cancer (OAC in Euro-America, squamous cell cancer in East Asia (32), or confounding by health behaviours. In an analysis among 922,896 patients in the Cancer Prevention Study-II, which included over 1,300 deaths from oesophageal cancer, increased coffee consumption was associated with increased cancer mortality (33). Furthermore, a recent UK Biobank study demonstrated a dose-response between number of cups of coffee and the squamous cell histological subtype of oesophageal cancer specifically, with an 8% increased risk reported per cup (28). A carcinogenic effect of coffee drinking in the oesophagus may therefore previously have been obscured by confounding or analysis of all subtypes together.

The potential mechanisms of coffee-induced oesophageal carcinogenesis include the detrimental effects of enhanced gastroesophageal reflux, which is known to occur with coffee intake and may promote inflammation. Caffeine is a key bioactive component of coffee and we provide weak evidence for it mediating the carcinogenic effects of coffee as individuals with a preference for caffeinated drinks had a higher risk than those with a preference for decaffeinated drinks. Although genetically-predicted caffeine consumption was found to be associated with GI cancer, oesophageal cancer and multiple myeloma to a similar extent as coffee, it is important to note that there was significant overlap in the genetic instruments for coffee and caffeine consumption, hence this should not be regarded as an independent analysis. The instrument for caffeine intake includes SNPs near genes with an established direct or indirect role in caffeine metabolism (i.e., CYP1A1 and AHR, the latter regulates the expression of CYP1A1) whereas the coffee consumption instrument includes a broader range of SNPs of which some are located in genes related to smell or taste perception of coffee or the rewarding response to caffeine (19). It should also be noted that genetically predicted higher caffeine intake is associated with lower plasma caffeine levels (34) Furthermore, anticarcinogenic effects of caffeine have previously been reported(5) and previous studies investigating caffeine intake and cancer have been null (35).

Interestingly, thermal injury to the oesophagus may also be an important oncogenesis driving factor. Consumption of very hot drinks, particularly mate, has been associated with an increased risk of oesophageal cancer (11). Furthermore, this phenomenon also applies to ingestion of hot foods (36,37), hot drinks can cause oesophagitis (38) which is a pre-cursor to cancer, and hot water has been found to promote carcinogenesis in rat and mouse models of oesophageal cancer (39)(40). It is therefore plausible that a carcinogenic effect of coffee relates to thermal injury broadly, rather than being specific to coffee or its constituents, as reflected in the IARC statement (11). We provide further evidence in support of the thermal injury hypothesis by demonstrating that oesophageal cancer risk is consistently augmented in all strata of hot beverage drinking. Although the precision was limited in the very hot strata due to low power, it is also notable that the magnitude of the effect was largest for individuals in the two strata with preference for the warmest drinks. Lastly, we found genetically predicted coffee consumption to be associated with oesophageal cancer to a similar extent in non-drinkers of coffee compared to in coffee drinkers with a preference for caffeinated drinks. Although counter-intuitive, this can be reconciled with previous evidence in UK Biobank that genetic risk scores for coffee consumption were positively associated with consumption of a range of beverages, including both caffeinated and decaffeinated coffee and tea, most subtypes of coffee and standard tea consumption (20). The strata of non-coffee drinkers amongst those with a high genetically predicted coffee consumption therefore likely represents consumers of warm tea. This underscores that the effect is likely related at least in part to temperature, and echoes the need for further research into this as a putative mechanism. When stratifying analyses by caffeine preference, associations were weaker for those with preference for decaffeinated coffee. This may be a chance finding as confidence intervals were wide, or it may be that the harmful effect of hot drink consumption is lower for non-caffeinated drinks than for caffeinated drinks. Alternatively, smoking prevalence was lower for those who preferred non-caffeinated drinks; it may be that the thermal effect of hot drink consumption is stronger in individuals who smoke regularly due to the damage to the oesophagus from smoking.

Coffee consumption has previously been reported to have a protective effect on multiple non-digestive system cancers (9)(41)(42). However, the present study did not find a linear causal association with the majority of cancer types studied. This is consistent with the previous literature on MR analyses of individual cancers of the prostate (15), colorectum (13), ovary (16) and breast (14) and of eight cancer types in the UK BioBank (12). Meta-analysis with ovarian cancer consortium data revealed a weak negative association with ovarian cancer (43), for which we provide some support. Lastly, the only non-digestive system cancer for which we found a positive causal association with coffee consumption was multiple myeloma in UK Biobank; but this was not replicated in FinnGen. In contrast a prospective study of participants of the Västerbotten Intervention Project showed an inverse relationship between coffee consumption and incident myeloma (44). A nested control of American Cancer Society volunteers (45) and The Japanese Public Health Center-based Prospective Study did not find an observational relationship with coffee consumption amongst the 138 cases of multiple myeloma included (46), but there are no previous MR analyses for the condition for comparison. Overall, the present study provides no evidence for either pro- or anticarcinogenic effects for the majority of cancer types, but further research is warranted building upon the associations we have found with multiple myeloma, oesophageal and ovarian cancers.

We present the most comprehensive assessment of the association of coffee consumption with a broad range of site-specific cancers based on the MR design, and the first for some cancers, including oesophageal cancer. Unlike previous MR analyses in the area, we account for pleiotropic effects of BMI and smoking, which are genetically correlated with coffee consumption, as well as alcohol consumption, which is not clearly genetically correlated with coffee consumption. Although MR studies minimize bias due to confounding, we cannot entirely rule out that other dietary factors known to be carcinogenic, such as red meat consumption, might be correlated with genetic predisposition to consume more coffee. We limited our analyses to individuals of European-descent to minimise population-structure biases but this impacts upon the generalisability of the results to other populations. Another important limitation is the use of UK Biobank for estimation of both exposure and outcome datasets, which resulted in slight participant overlap with the potential for weak instrument bias, although this is mitigated by a relatively high F-statistic. Furthermore, coffee consumption varies throughout the lifetime, but as genetic variants are fixed at conception and our MR analyses estimated lifelong coffee consumption, we were unable to assess the risk of coffee consumption at different stages of life in relation to cancer risk. Moreover, our findings for oesophageal cancer and multiple myeloma were directionally consistent in an independent sample with no overlap with the coffee GWAS meta-analysis. Reduced power from low case numbers for some cancer types resulted in low precision estimates. Lastly, we were unable to assess for possible U- or J-shaped associations between coffee consumption and cancer risk.

## Conclusions

In conclusion, we provide evidence for coffee consumption being causally associated with risk of oesophageal cancer with some evidence this is related to a temperature effect. Otherwise, our results do not support a linear causal association with the majority of cancer types studied, other than limited evidence for harmful and protective associations with multiple myeloma and ovarian cancers respectively. As this field is acknowledged by expert international bodies to be understudied, it is imperative that further studies explore these relationships and the possible mechanisms of coffee consumption in oesophageal carcinogenesis.

## Supplementary Material

Supplementary tables

## Figures and Tables

**Fig 1:**
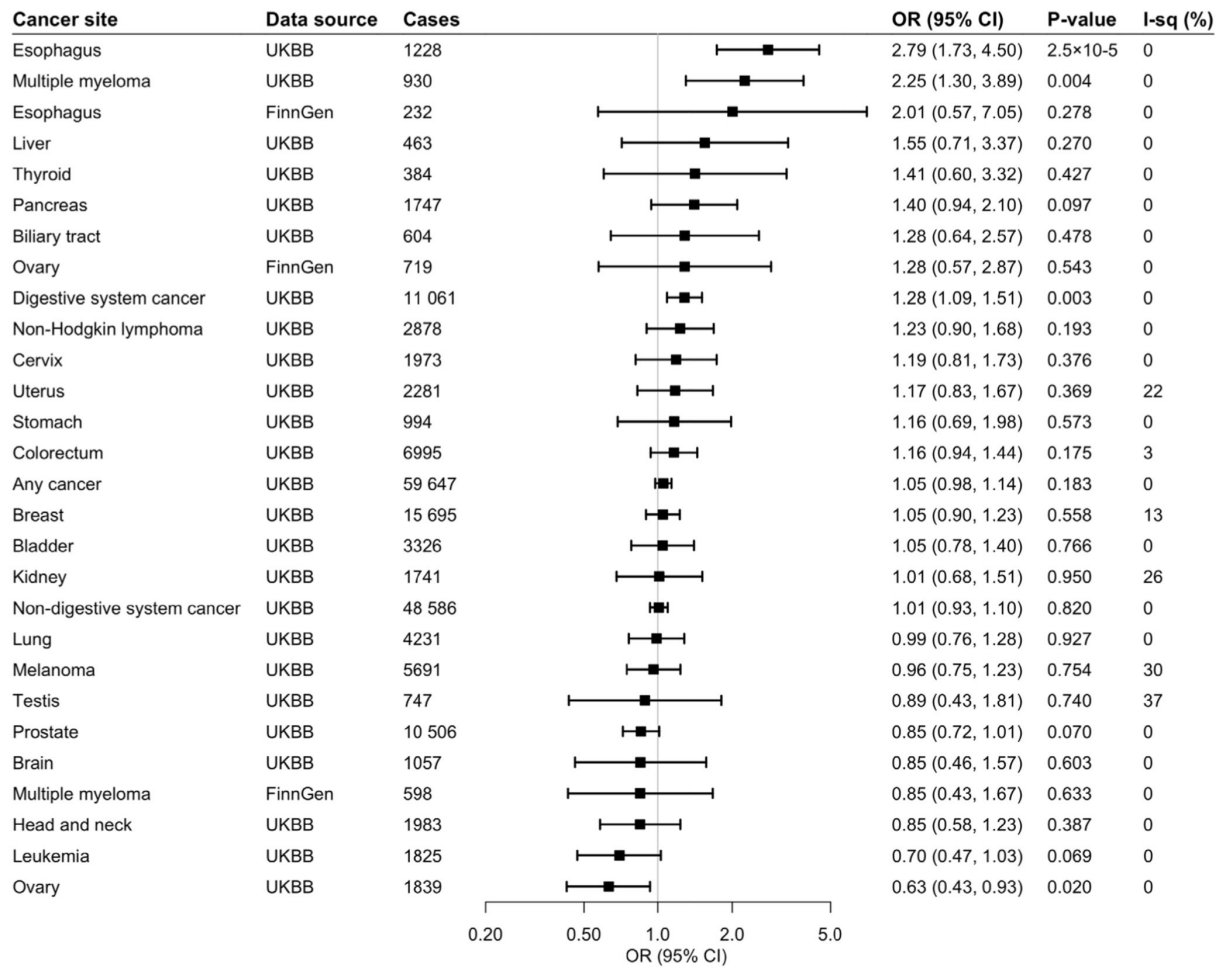
Associations of genetic predisposition to coffee consumption with site-specific cancers. Odds ratios are per 50% increase in coffee consumption. Results are obtained from the inverse-variance weighted median method with random-effects model. The I^2^ statistic quantifies the amount of heterogeneity among estimates based on individual SNPs. UKBB, UK Biobank; CI, confidence interval; OR, odds ratio.

**Fig 2:**
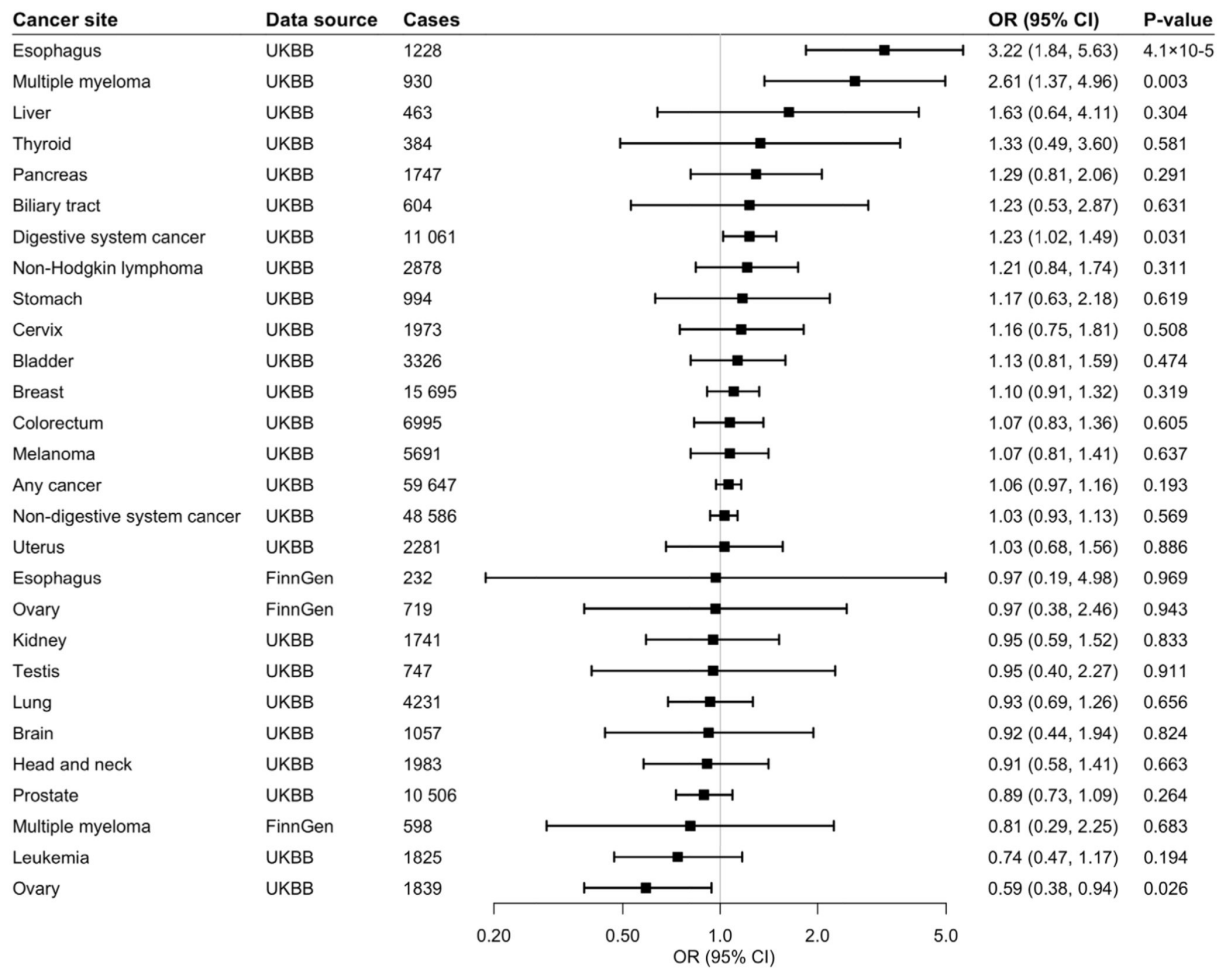
Associations of genetic predisposition to coffee consumption with site-specific cancers with adjustment for BMI. Odds ratios are per 50% increase in coffee consumption after adjustment for genetically predicted BMI. Results are obtained from the inverse-variance weighted median method with random-effects model. BMI, Body Mass Index; UKBB, UK Biobank; CI, confidence interval; OR, odds ratio.

**Fig 3:**
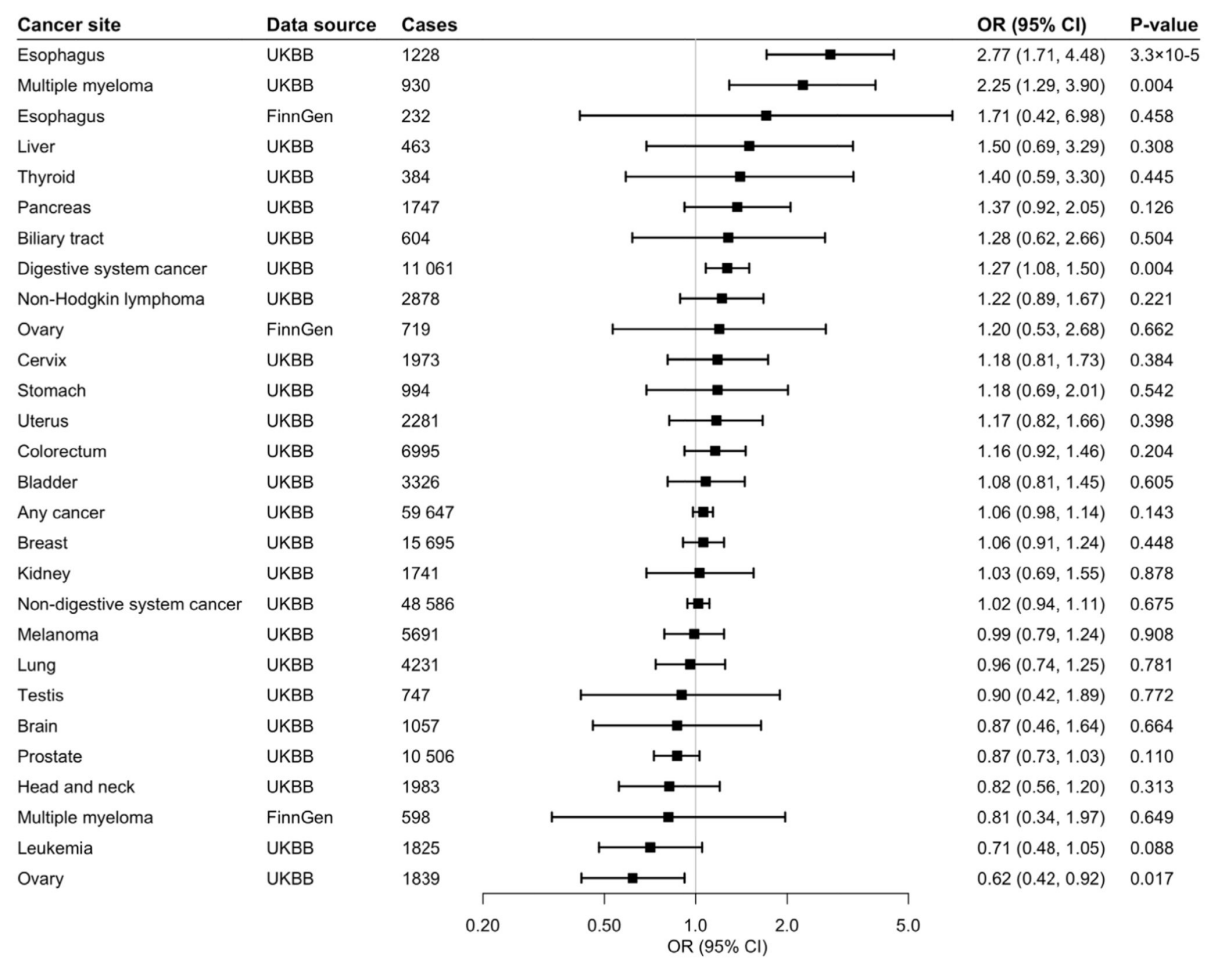
Associations of genetic predisposition to coffee consumption with site-specific cancers with adjustment for smoking initiation. Odds ratios are per 50% increase in coffee consumption after adjustment for genetic predisposition to smoking initiation. Results are obtained from the inverse-variance weighted median method with random-effects model. based on individual SNPs. UKBB, UK Biobank; CI, confidence interval; OR, odds ratio.

**Fig 4:**
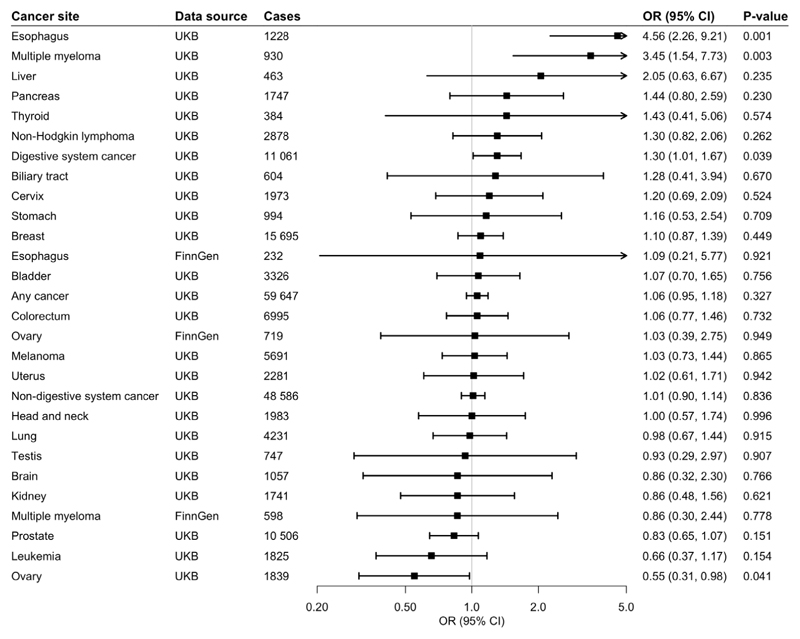
Associations of genetic predisposition to coffee consumption with site-specific cancers with adjustment for both BMI and smoking initiation. Odds ratios are per 50% increase in coffee consumption after adjustment for genetic predisposition to BMI and smoking initiation. Results are obtained from the inverse-variance weighted median method with random-effects model. UKBB, UK Biobank; CI, confidence interval; OR, odds ratio.

**Fig 5:**
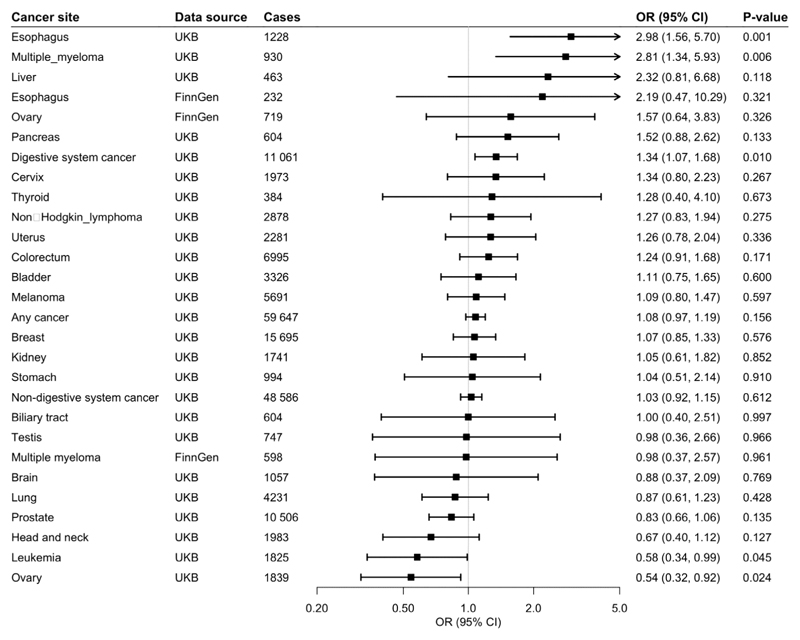
Associations of genetic predisposition to coffee consumption with site-specific cancers with adjustment for alcohol consumption. Odds ratios are per 50% increase in coffee consumption after adjustment for genetic predisposition to alcohol consumption. Results are obtained from the inverse-variance weighted median method with random-effects model.. UKBB, UK Biobank; CI, confidence interval; OR, odds ratio.

**Fig 6:**
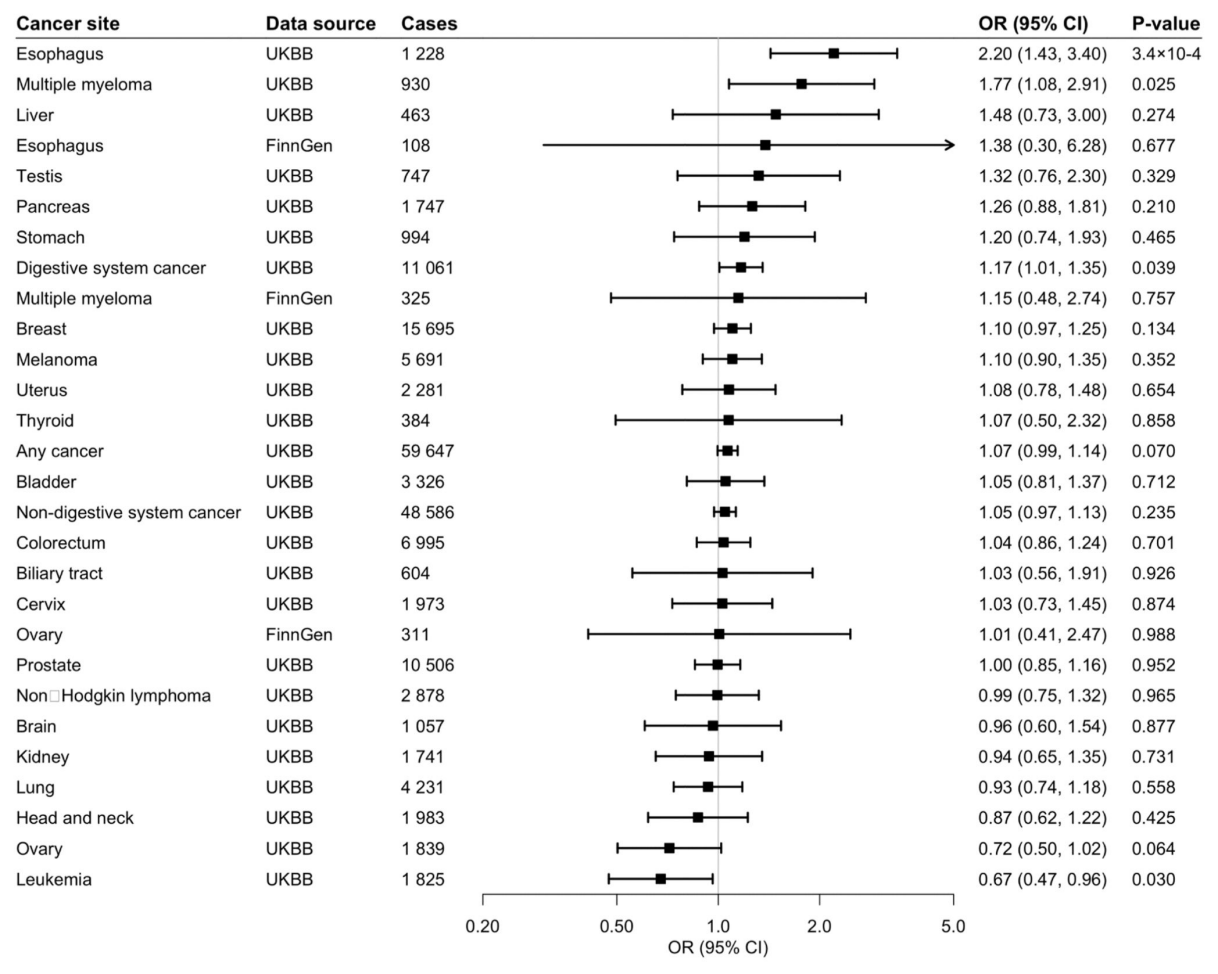
Associations of genetic predisposition to caffeine consumption with site-specific cancers. Odds ratios are per 50% increase in caffeine consumption. Results are obtained from the inverse-variance weighted median method with fixed-effects model. UKBB, UK Biobank; CI, confidence interval; OR, odds ratio.

**Table 1:** Genetic instruments for coffee and caffeine consumption

SNP	Chr	Pos (hg_37)	Nearby gene	EA	NEA	EAF	Beta	SE	P value
Coffee Consumption									
rs574367	1	177873210	*SEC16B*	T	G	0.21	0.021	0.004	8.06E-09
rs10865548	2	631606	*TMEM18*	G	A	0.83	0.031	0.004	4.46E-15
rs1260326	2	27730940	*GCKR*	C	T	0.61	0.027	0.003	2.62E-19
rs1057868	7	75615006	*POR*	T	C	0.29	0.039	0.003	5.26E-33
rs34060476	7	73037956	*MLXIPL*	G	A	0.13	0.038	0.004	5.06E-18
rs4410790	7	17284577	*AHR*	C	T	0.63	0.079	0.003	5.59E-141
rs73073176	7	17562952	*LOC101927630*	C	T	0.87	0.046	0.004	5.56E-25
rs597045	11	56272114	*0R8U8*	A	T	0.69	0.021	0.003	6.62E-11
rs1956218	14	33075243	*AKAP6*	G	A	0.56	0.016	0.003	3.62E-08
rs2472297	15	75027880	*CYP1A1/2*	T	C	0.27	0.091	0.003	5.19E-155
rs66723169	18	57808978	*MC4R*	A	C	0.23	0.029	0.004	9.88E-17
rs2330783	22	24747031	*SPECC1L-ADORA2A*	G	T	0.99	0.091	0.013	1.57E-12
Caffeine Consumption									
rs4410790	7	17244953	*AHR*	C	T	0.62	0.150	0.017	2.36E-19
rs2470893	15	74727108	*CYP1A1*	T	C	0.31	0.120	0.016	5.15E-14

Chr, chromosome; EA, effect allele; EAF, effect allele frequency; NEA, non-effect allele; Pos, position; SNP, single nucleotide polymorphism. Beta and SE were scaled to 50% increase in coffee consumption.

## Data Availability

The data that support the findings of this study are available from the corresponding author, SL, upon reasonable request.
